# Facile Fabrication of Multi-hierarchical Porous Polyaniline Composite as Pressure Sensor and Gas Sensor with Adjustable Sensitivity

**DOI:** 10.1186/s11671-017-2246-y

**Published:** 2017-08-01

**Authors:** Xiao-Xiao He, Jin-Tao Li, Xian-Sheng Jia, Lu Tong, Xiao-Xiong Wang, Jun Zhang, Jie Zheng, Xin Ning, Yun-Ze Long

**Affiliations:** 1 Collaborative Innovation Center for Nanomaterials & Devices, College of Physics, Qingdao University, Qingdao, 266071 China; 2 Industrial Research Institute of Nonwovens & Technical Textiles, Qingdao University, Qingdao, 266071 China

**Keywords:** Facile fabrication, Low cost, PANI composite, Flexible pressure sensor, Adjustable sensitivity gas sensor

## Abstract

A multi-hierarchical porous polyaniline (PANI) composite which could be used in good performance pressure sensor and adjustable sensitivity gas sensor has been fabricated by a facile in situ polymerization. Commercial grade sponge was utilized as a template scaffold to deposit PANI via in situ polymerization. With abundant interconnected pores throughout the whole structure, the sponge provided sufficient surface for the growth of PANI nanobranches. The flexible porous structure helped the composite to show high performance in pressure detection with fast response and favorable recoverability and gas detection with adjustable sensitivity. The sensing mechanism of the PANI/sponge-based flexible sensor has also been discussed. The results indicate that this work provides a feasible approach to fabricate efficient sensors with advantages of low cost, facile preparation, and easy signal collection.

## Background

Nowadays, varieties of sensors, including pressure sensor [1, 2], strain sensor [3, 4], gas sensor [5–7], temperature sensor [8, 9], and displacement sensor [10], have been extensively explored. Particularly, with the popularity of artificial intelligence technology, low-cost flexible sensors are highly desirable for the fabrication of portable, wearable, and foldable devices. However, it is usually expensive and complicated to design flexible sensors with elaborate structures [11, 12]. Thus, an efficient and low-cost approach is highly required to fulfill flexible and portable sensors.

Sponge, as a three-dimensional (3D) ubiquitous material, has aroused extensive concerns due to its unique performance, such as high elasticity, high specific surface area, low density, and low-cost manufacturing. Therefore, conductive sponges are considered as excellent materials to assemble sensors and devices, such as graphene-polyurethane sponge as pressure sensor [13], superhydrophobic polyaniline (PANI) sponge as oil absorbent [14], and graphene platelets/PANI sponge [15] as supercapacitors. Herein, besides carbon series semiconductor materials, conducting polymer is often used as functional element of devices due to their good electrical conductivity, physical robustness, and large surface area [3, 16, 17]. As one of conducting polymers, for the aim of fabricating flexible and low-cost sensors, PANI has already been used as sensing material in various application fields, such as supercapacitors [18, 19], sensors [3, 20], electrodes [21, 22], microwave absorption [23], and electromagnetic shielding [24]. In general, there are two main methods to prepare PANI composites: doping and in situ polymerization [3, 25–27]. Normally, in situ polymerization provides more feasible preparation and remarkable effectiveness.

Generally, for pressure sensors, according to the sensing mechanisms, there mainly exist piezoelectric sensors [28, 29], capacitive sensors [30], transistor sensors [2, 31], and piezoresistive sensors [13, 32, 33]. Piezoresistive sensor, as a typical pressure sensor, which transduces pressure to resistance signal, has been widely used due to outstanding advantages, such as simple principle, convenient signal collection, low cost, and simple preparation [13, 28, 32, 33]. Additionally, for gas sensor, the alkali gas sensing mechanism of PANI can be attributed to the conducting mechanism [20]. As we know, the charge carriers of PANI are polarons, and the conjugated molecule chain in PANI will become more conductive after the doping of proton. When the alkaline gas molecules are absorbed by the nanostructured PANI, this will result in a decrease of the charge carriers and increase of the electrical resistance of PANI.

In this study, we used in situ polymerization method to prepare multi-hierarchical porous PANI/sponge composite for piezoresistive sensor and adjustable sensitivity gas sensor. As a porous scaffold, the sponge provided sufficient surface for the growth of nanostructured PANI. The sensor with abundant pores and PANI nanostructures showed excellent performances in pressure sensitivity with fast response to diverse pressure and release. The mechanism of piezoresistive sensing could be attributed to the resistance change by the contact variation of the conductive porous structure. Besides, based on the conducting mechanism of PANI and the piezoresistive sensing mechanism mentioned above, the potential application of the composite for adjustable sensitivity gas sensor has also been investigated. The results indicate that this work provides an effective and low-cost approach to fabricate porous conductive composite and device.

## Methods

### Materials

Ammonium persulfate (APS, *M*_*w*_ = 228.20), 5-sulfosalicylic acid (SSA, *M*_*w*_ = 254.22), and ammonia solution were supplied by Sinopharm Chemical Reagent Co., Ltd. (Shanghai China). The aniline (*M*_*w*_ = 93.13) was purchased from Chemical Reagent (Tianjin China). The sponge was commercial grade polyurethane sponge (Brand: Domaxe, China).

### Preparation of PANI/Sponge Composite

**Fig. 1:**
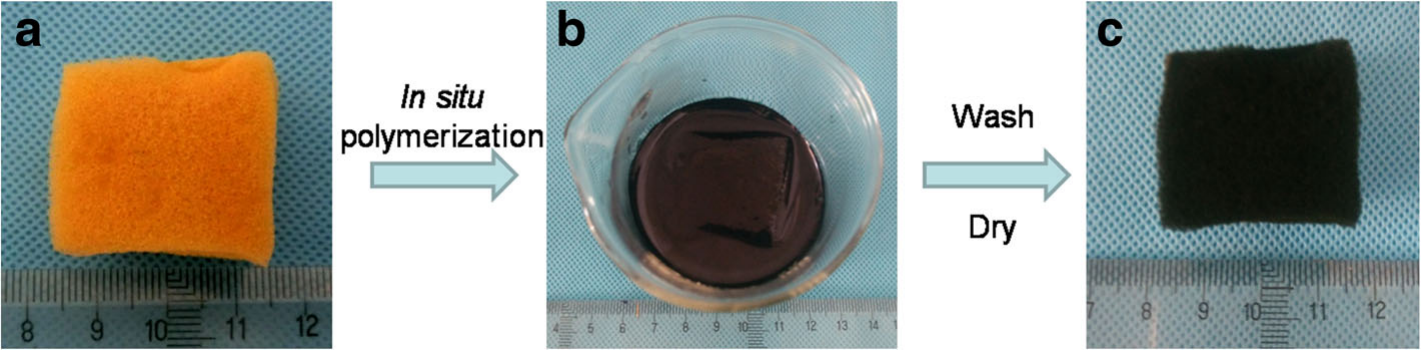
The process of preparing PANI/sponge composite. **a** A commercial grade polyurethane sponge was selected. **b** In situ polymerization of PANI on the sponge. **c** The sample was washed with DI water and dried at room temperature to obtain the final PANI/sponge composite

In situ polymerization method was used to prepare PANI/sponge composite. Briefly speaking, 2.5422 g of SSA and 1.8626 g of aniline were well dispersed in 50 ml of deionized (DI) water with magnetic stirring for 20 min. Then, the sponge, which was regarded as scaffold, was submerged in the prepared solution. After that, APS solution (4.5640 g of APS in 50 ml of DI water) was slowly added to the above solution to make sure the uniform and intensive mixing. After 24 h’ standing in refrigerator at 2 °C, the sponge was taken out from the final solution and washed with DI water to remove the impurities. Drying at room temperature for 48 h, the PANI/sponge composite was finally obtained. As seen in Fig. 1, the sample (sponge) underwent a color change from yellow to deep green (PANI/sponge). The shape and volume of final PANI/sponge were unchanged because of the strength and toughness of scaffold; 35% of PANI mass load was evaluated by contrasting the weight of sponge and PANI/sponge composite.

### Sensor Assembly

**Fig. 2:**
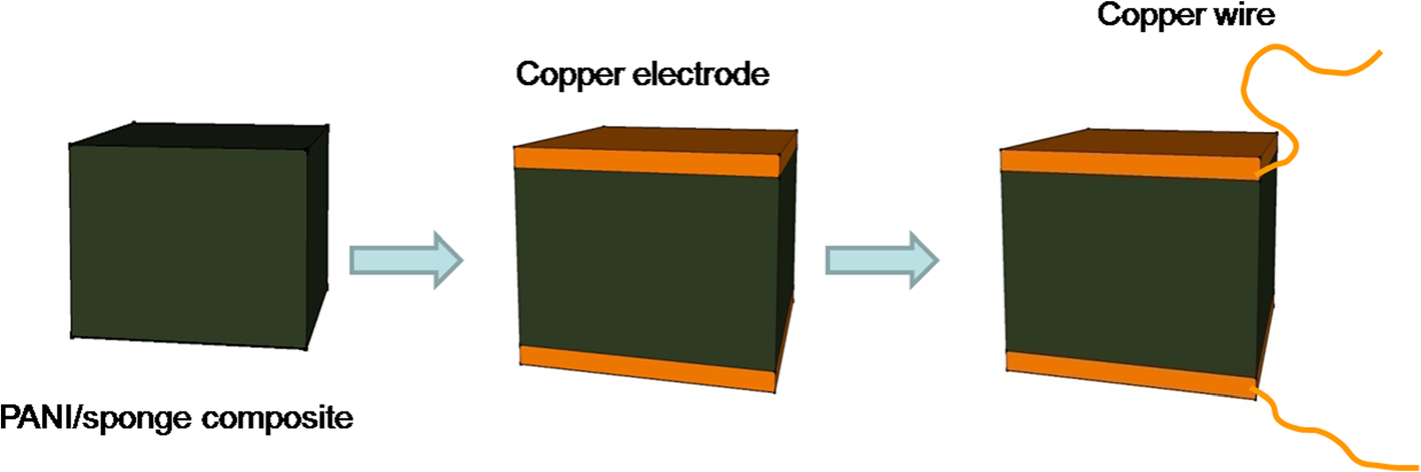
Schematic of the preparation of PANI/sponge sensor

As shown in Fig. 2, a simple piezoresistive sensor was assembled by sandwiching PANI/sponge composite between two copper electrodes (copper sheet), and the size of the composite was 2 × 2 × 2 cm^3^. Two copper wires were fixed on the copper electrode by soldering tin. The copper wires were used to connect with electrical property measurement system, which could response to various pressures applied on the sensor.

### Characterization

The sponge and PANI/sponge composite were characterized by a scanning electron microscope (SEM, JEOL, JSM-7500F) and a Micro-Roman spectroscopy system (Renishaw inVia Plus, 50 mW DPSS laser at 532 nm). The electrical properties were measured by a Keithley 6487 high-resistance meter system.

## Results and Discussion

### Morphological and Structural Properties

**Fig. 3:**
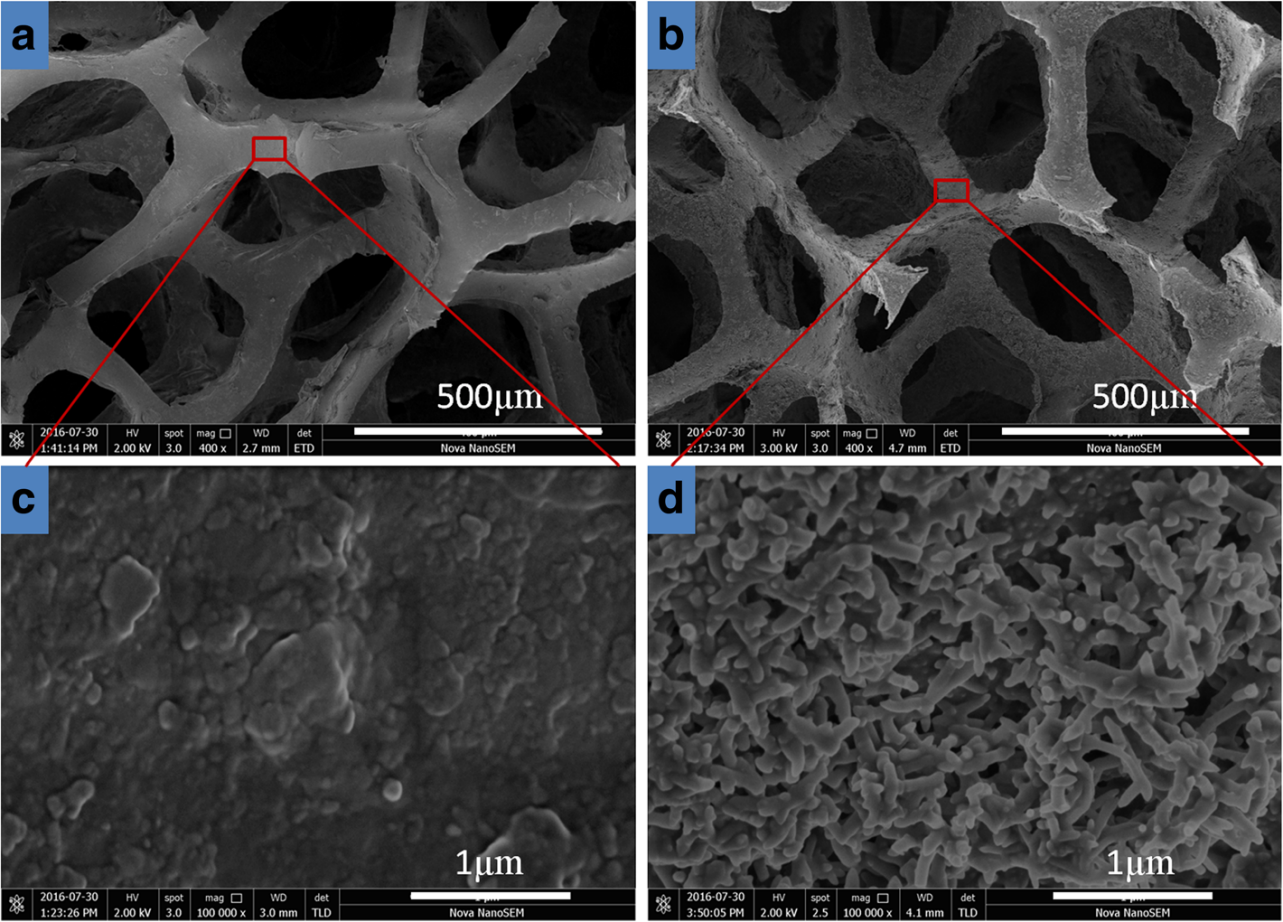
SEM images of **a**, **c** pristine sponge and **b**, **d** sponge after in situ polymerization

Figure 3a, c and Fig. 3b, d show SEM images of pristine sponge and in situ polymerized sponge under different magnifications, respectively. It can be seen that the interconnected porous structure provides sufficient surface for the growth of PANI nanobranches. The composite after polymerization exhibits rough surface while the pristine sponge is smooth, which indicates that PANI micro/nanostructures have grown. Under high magnification, the PANI nanobranches could be seen clearly on the surface of sponge. During the in situ polymerization process, due to intrinsic non-uniformity of PANI, some bumps are generated in PANI membrane [27], and then, PANI nanobranches could in situ grow on the sponge structure with adequate adhesion by interfacial compatibility. The nanostructured PANI coating helps the composite to improve its electrical conductivity. Meanwhile, the special nanobranches make the composite a bigger specific surface area, so that the composite may display excellent properties in some contact-dependent applications. Moreover, this PANI/sponge composite has an interesting multi-hierarchical porous structure, which is comprised of the sponge with micropores (Fig. 3b) and the PANI branches with nano-pores (Fig. 3d).

### Raman Spectra

**Fig. 4:**
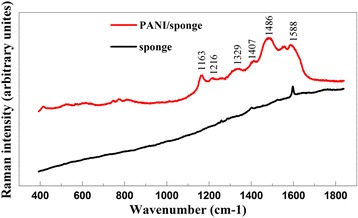
Raman spectrum of pristine sponge and sponge after in situ polymerization

The Raman spectra of the pristine sponge and PANI/sponge composite are depicted in Fig. 4. According to the characteristic peak positions of PANI/sponge composite, the spectra exhibit most of the characteristics of PANI. The band around 1486, 1407, 1216, and 1163 cm^−1^ are assigned to quinondiimine. Band 1486 cm^−1^ corresponds to C=C and C=N allied stretching vibration, band 1407 and 1216 cm^−1^ correspond to C–N stretching vibration, and band 1163 cm^−1^ corresponds to C–N bending vibration, respectively. Besides, the band at 1329 cm^−1^ represents C–N stretching vibration of phenylenediamine. The band around 1588 cm^−1^ is assigned to C–C stretching vibration (corresponding region is from 1550 to 1650 cm^−1^). The results confirm the successful polymerization and the existence of PANI on sponge.

### Pressure Sensitivity Test

To demonstrate pressure sensitivity, the resistance variation of the PANI/sponge composite with pressure applied on surface was explored. The composite with 3D size of 2 × 2 × 2 cm^3^ was sandwiched by two copper electrodes (as shown in Fig. 2), and the electricity was recorded with the applying of pressure on the two electrodes.

**Fig. 5:**
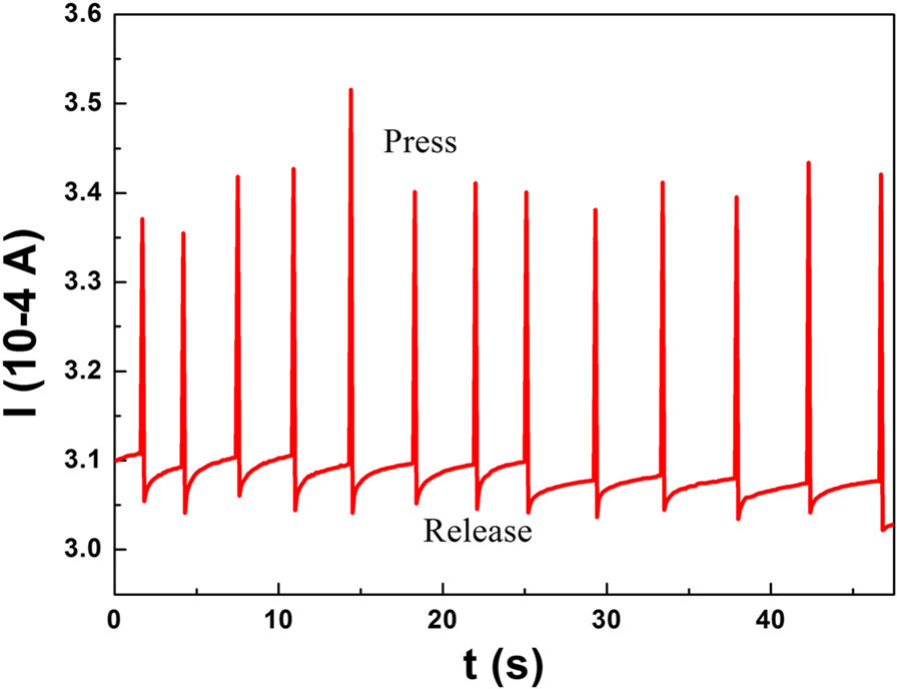
Cyclic pressure-removed response of the PANI/sponge with about 2-mm compressive deformation forced by finger

**Fig. 6:**
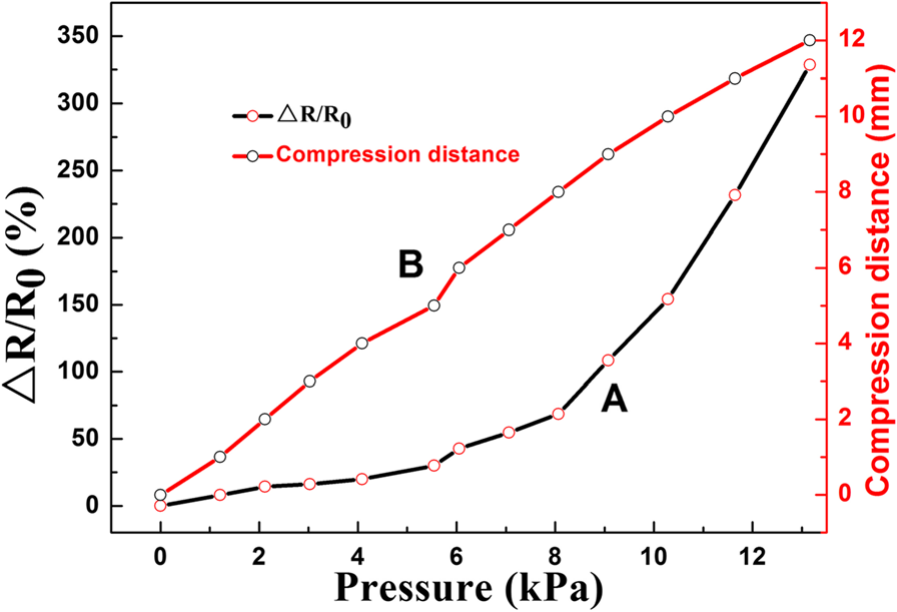
*A* Pressure-response curve of the PANI/sponge sensor and *B* the corresponding relationship curve of pressure and compression deformation

Firstly, a simple exploration is carried out by a cyclic pressure-removed response (Fig. 5) of the PANI/sponge sensor at a fixed bias of 5 V, and there is about 2-mm compressive deformation forced by finger. As shown in Fig. 5, the current reaches to peak value fast with the applying of pressure, and as release, it could recover to initial value immediately and stays in a good stability. Meanwhile, the sensibility and recoverability are not affected by multiple press-release cycles. On the other hand, the peaks are not uniform, which may be caused by the small fluctuations of compression deformations for the press of human finger is not absolutely uniform. To systematically demonstrate the sensitivity of PANI/sponge to different pressures, the electronic resistance variation ratios calculated on the basis of measured data are shown in Fig. 6 (a). Here, Δ*R*/*R*_0_ = (*R*_0_ − *R*)/*R*_0_, where *R*_0_ and *R* denote the resistance in release and pressure condition. It can be seen that the relative change of resistance is increased when the PANI/sponge is pressed from 0 to 13 kPa. Furthermore, from the slope of curve A, the pressure sensitivity *S* (*S* = *δ*(Δ*R*/*R*_0_)/*δP*, where *P* denote the applied pressure) [13], which is an important index to reflect the performance of a pressure sensor, could be calculated to be about 8.0 (0–8 kPa) and about 54.5 (8–13 kPa). We confirm that the sensing mechanism of PANI/sponge composite is the change of inner microporous structure. Here, for easily operation, compression distance is proposed to characterize the strength of the applied pressures, and the corresponding relationship of pressure and compression deformation is illustrated in Fig. 6 (b).

**Fig. 7:**
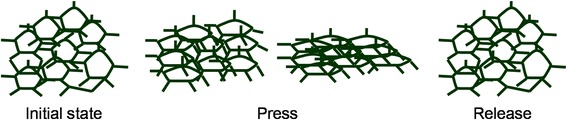
Pressure-sensing schematic of PANI/sponge composite

**Fig. 8:**
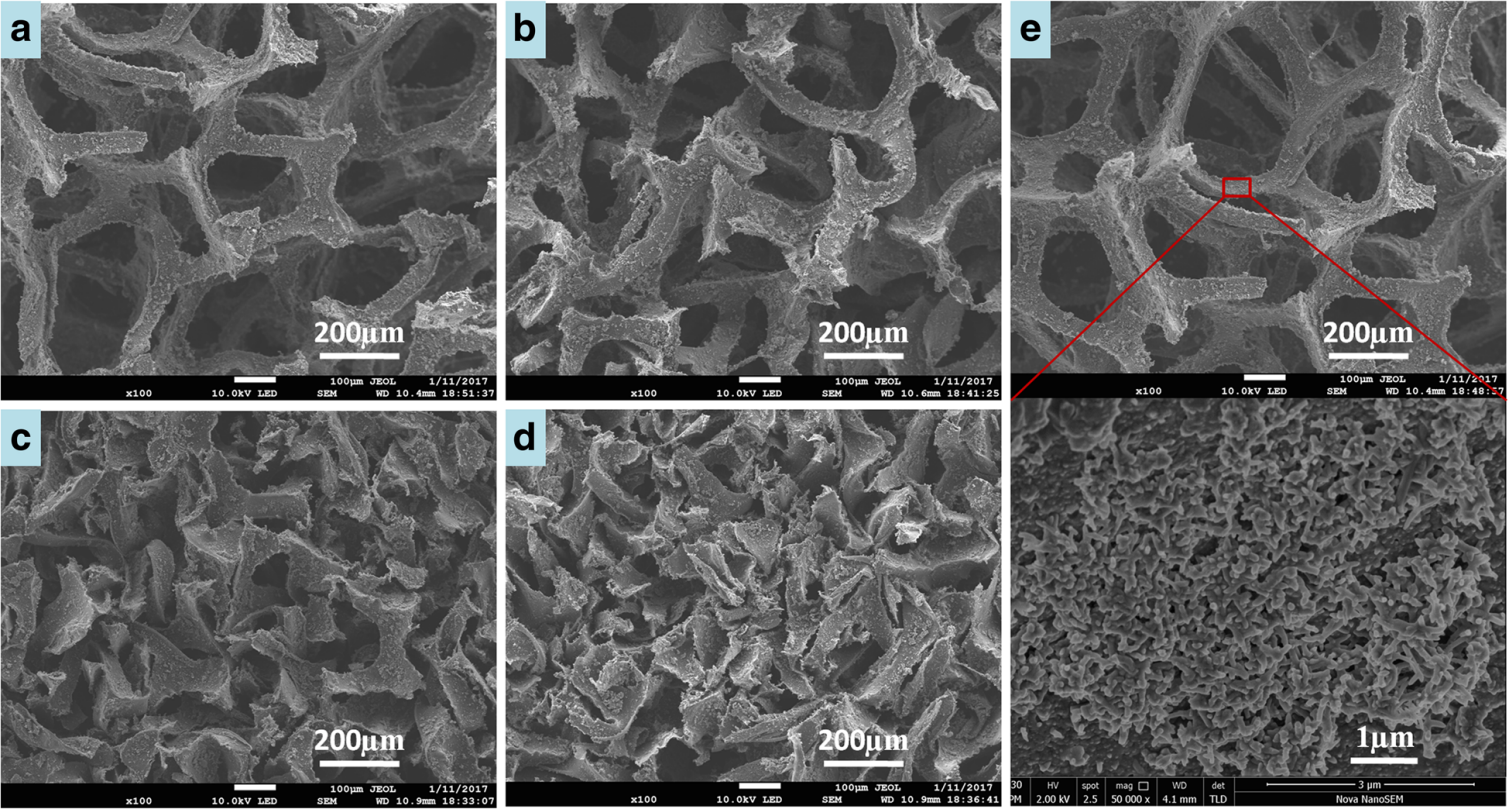
SEM images of microporous structure of PANI/sponge composite under different pressures with approximate compression ratio of **a** 0%, **b** 20%, **c** 40%, and **d** 60%. **e** SEM images of the composite after multiple pressures under different magnifications

To demonstrate the piezoresistive sensing mechanism of the conductive PANI/sponge composite, a simple schematic diagram (Fig. 7) is depicted to simulate the microporous contact change of the sponge structure. With the increase of pressure, the micropores get squashed and contact each other more closely. Particularly, the microporous structure could recover to previous condition with the release of pressure. Herein, the resistance gets smaller with the increase of pressure and could return to initial value after release. Thus, the inner-contact variation of the conductive porous structure results in the resistance change, which generates the piezoresistive sensitivity. To illustrate the contact variation visually, SEM images of microporous structure under different degrees of pressure are shown in Fig. 8a–d. Besides, there is no PANI desquamation in testing, as demonstrated in Fig. 8e (SEM images of the composite after multiple pressures), the PANI micro/nanostructures could maintain adequate adhesion to the sponge after cyclic testing.

**Fig. 9:**
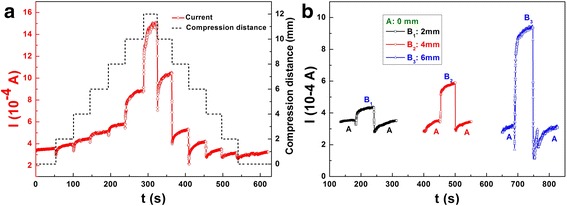
Stability and recoverability test of the PANI/sponge sensor. **a** Current responses to different pressures with compressive deformation from 0 to 12 mm and back to 0 mm. **b** Current responses to loading and unloading pressure with different intensities

A pressure sensor should be equipped with capabilities of good stability and recoverability. For the purpose of demonstrating the stability and recoverability characteristics, the current responses to different pressures under a fixed bias of 5 V are tested. As exhibited in Fig. 9a, the current almost displays a liner response to compression deformation from 0 to 12 mm and back to 0 mm; meanwhile, it holds a fast response and a good stability to the ascending and descending pressures, besides, there only exists a little deviation between a continuous ascending and descending test. However, there arises a clear difference between 250~300 s and 320~360 s. We infer that this deviation may be caused by two main reasons. One is that there may be a hysteresis quality when the composite is suddenly recovered from the biggest deformation. The other is the possible operating error in testing, which leads to a bigger compression distance than that in 250~300 s. To characterize the stability and recoverability more directly, Fig. 9b demonstrates the current responses to loading and unloading pressure with different intensities. From the circle response curves, the composite responses to the pressures immediately, and the current could fully recover to the initial value within 35 s after withdrawing the pressure. It can be seen from Fig. 9 that the current increases with increasing pressure and decreases with decreasing pressure, which is consistent with the piezoresistive sensing mechanism illustrated in the above. These results indicate that the flexible and sensitive PANI/sponge composite is potentially applicable in pressure sensors, which may be used in low-cost artificial skin and smart clothing [13, 34, 1].

### Application in Finger Bending-Release Detection

**Fig. 10:**
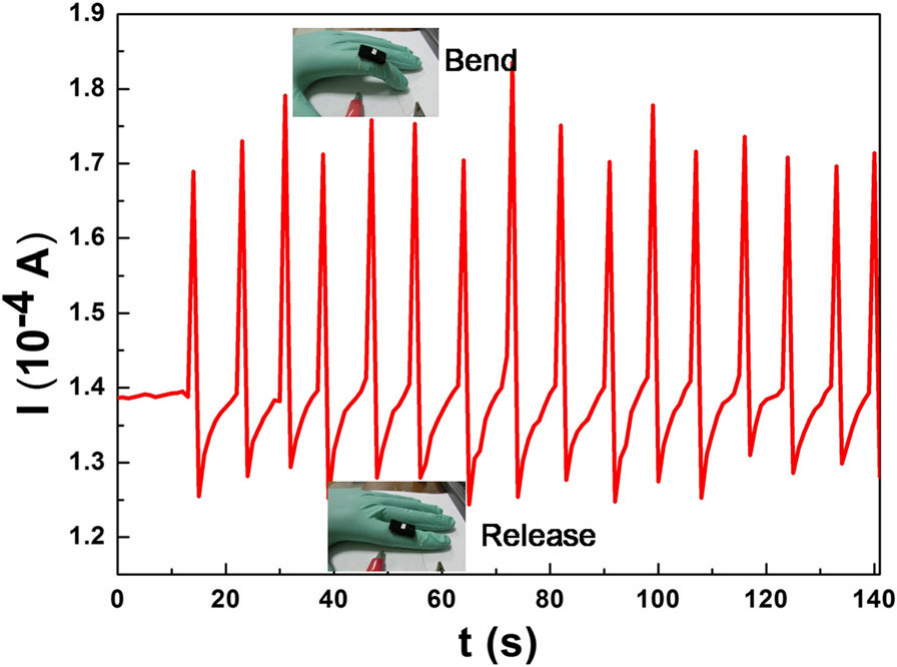
Current responses of finger bending-release motion detection at a fixed bias of 5 V

Nowadays, low-cost pressure sensors with high sensitivity and proper flexibility are highly desirable in portable and wearable devices. Here, the simple PANI/sponge sensor (2 × 1 × 0.5 cm^3^) is fixed on a rubber glove to the joint of forefinger. The current response is recorded while the tester performs finger bend-release operations at the fixed bias of 5 V. Several cycle current responses are shown in Fig. 10. The finger bends and releases rapidly in this process. It is noticed that the current increases sharply when the finger is suddenly bent. When the finger is released, the current exhibits a significant reduction and recovers to its original value. The degrees of every finger bending are not exactly same, so the current peaks at each bend point have a little difference. The sensibility and repeatability of current responses indicate that the sensor is reliable and capable for flexible detection devices in some low-cost portable and wearable devices.

### Application in Adjustable Sensitivity Gas Sensor

**Fig. 11:**
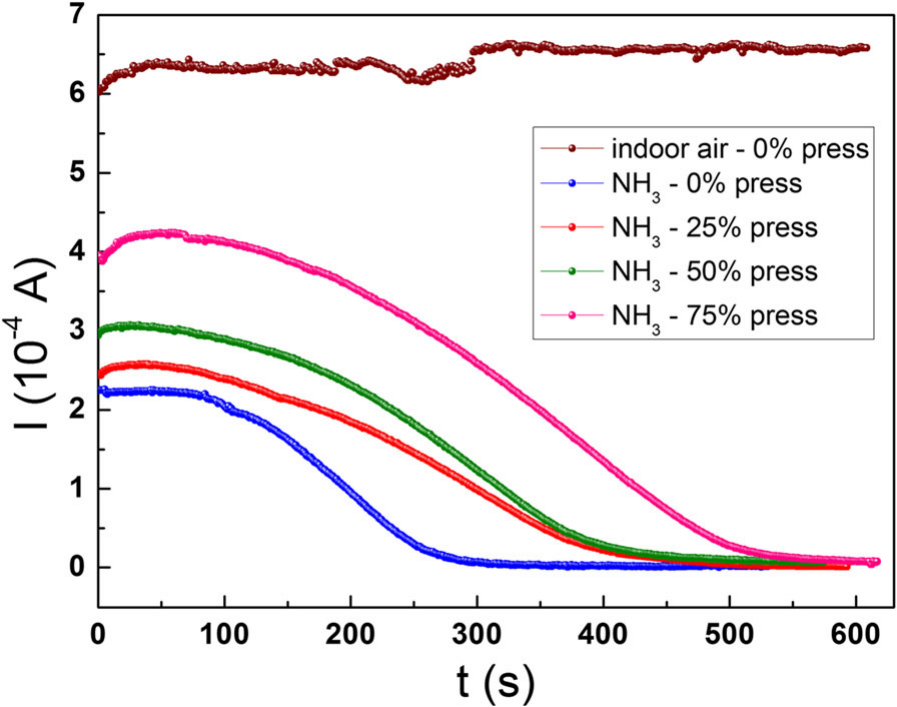
NH_3_ sensing properties of the PANI/sponge composite under different pressures

PANI composites have been widely explored as gas sensing materials for their unique conducting mechanism. However, the related reports on PANI-based gas sensor mainly focus on a fixed or a single sensitivity. Herein, based on the flexible porous structure and the reaction of NH_3_ molecules with proton-doped PANI, we investigate the potential application of PANI/sponge composite on adjustable sensitivity NH_3_ gas sensor. Through controlling the inner-contact density of the conductive porous structure (as shown in Fig. 8), the diffusion volume and rate of air inflow can be adjusted to achieve the purpose of adjustable sensitivity. The sandwiched PANI/sponge composite sensor under different pressures was put in a closed box (with size of 30 × 30 × 30 cm^3^) and contacted with the outside Keithley 6487 high-resistance meter system through copper wire. NH_3_ was produced by the natural volatilization of 1 ml ammonia solution added in box. Figure 11 exhibits the real-time PANI/sponge composite response to indoor air and NH_3_, which indicates that the compression degree effects on the sensitivity of NH_3_ detection. From the current-time (*I*-*t*) curves, it can be seen that the composite resistances with the diffusion of NH_3_ are obviously higher than that in indoor air. Besides, it is obvious that as the increase of compression degree, the composite resistance and the response time to steady state are both increased gradually under the same NH_3_ atmosphere, which indicates that the sensitivity could be adjusted by the inner contact porosity. As the increase of pressure, the inner-contact density of the conductive porous structure is increased, which leads to a decrease of both the diffusion volume and diffusion rate of NH_3_ inflow; therefore, under the same concentration, the response time to NH_3_ is extended. Moreover, the initial current increases with the increase of pressure due to the decreased inflow rate of NH_3_. On the other hand, because the content of NH_3_ in the closed box is the same, the current of the composite could reach a small value eventually, namely, dedoping of PANI by NH_3_ would reach a similar level.

## Conclusions

In conclusion, we report a facile method via in situ polymerization to prepare PANI/sponge composite which could be used in good performance pressure sensor and adjustable sensitivity gas sensor. The flexible interconnected porous structure helped the composite to show good sensitivity and recoverability to pressure. Besides, the flexible sensor based on PANI/sponge showed good performance in finger bending detection and NH_3_ detection with adjustable sensitivity. This work may provide a feasible approach to fabricate efficient portable and wearable devices with the advantages of low cost, facile preparation, and easy signal collection.
